# Design of the Prospective Real-world Outcomes Study of hepatic encephalopathy Patients’ Experience on Rifaximin-α (PROSPER): an observational study among 550 patients

**DOI:** 10.1186/s41124-017-0029-9

**Published:** 2018-01-08

**Authors:** Aleksander Krag, Marcus Schuchmann, Hanna Sodatonou, Jeff Pilot, James Whitehouse, Simone I. Strasser, Mark Hudson

**Affiliations:** 1 Department of Gastroenterology and Hepatology, Odense University Hospital, University of Southern Denmark, Odense, Denmark; 2 Department of Internal Medicine, Constance Medical Centre, Constance, Germany; 3 Norgine Ltd Clinical Development & Medical Affairs, Harefield, UK; 4 Norgine Global Health Outcomes, Norgine Ltd, Uxbridge, UK; 5 AW Morrow Gastroenterology and Liver Centre, Royal Prince Alfred Hospital and University of Sydney, Sydney, Australia; 6 Liver Unit, Freeman Hospital, Newcastle upon Tyne Hospitals NHS Trust and Institute of Cellular Medicine, Newcastle University, Newcastle upon Tyne, UK

**Keywords:** Cirrhosis, Health economics, Hepatic encephalopathy, Hospitalisation, Liver disease, Patient-reported outcomes, Quality of life, Real-world, Rifaximin-α 550 mg, Work productivity

## Abstract

**Background:**

Hepatic encephalopathy (HE) is one of the most important severe complications of liver cirrhosis. Thought to be caused by elevated blood levels of gut-derived neurotoxins (particularly ammonia) entering the brain, HE manifests as a wide range of neurological or psychiatric abnormalities, which increase the risk of mortality, result in substantial morbidity and negatively affect the quality of life (QoL) of both patients and their caregivers. HE is also associated with a substantial economic burden. Rifaximin-α 550 mg is a locally acting oral antibiotic that reduces the effects of ammonia-producing intestinal flora, and which is used to help reduce the recurrence of overt HE. The efficacy of rifaximin-α 550 mg was established in a randomised controlled trial and long-term extension study. However, ‘real-world’ evidence is also required to assess how this efficacy may translate into effectiveness in clinical practice, including the potential impact of treatment on healthcare resource utilisation.

**Methods:**

The Prospective Real-world Outcomes Study of HE Patients’ Experience on Rifaximin-α 550 mg (PROSPER) is a multinational, multicentre, observational study that will be conducted under real-world clinical practice conditions. Comprising a retrospective phase (up to 12 months) and a prospective phase (up to 24 months), and employing a robust statistical methodology, PROSPER has been specifically designed to minimise the bias associated with observational studies. The primary endpoint will be the effect of rifaximin-α 550 mg treatment on HE- and liver-related hospitalisation rate and duration of hospitalisation. Secondary endpoints will include comprehensive assessments of the impact of treatment on the QoL and workplace productivity of patients and caregivers, a global assessment of treatment effectiveness and safety/tolerability. Approximately 550 patients will be enrolled.

**Conclusions:**

PROSPER will provide valuable real-world information on the effectiveness of rifaximin-α 550 mg in reducing the recurrence of HE, and its impact on the QoL and work productivity of patients and their caregivers. By providing data on both the direct costs (e.g., hospitalisation rate, duration of hospitalisation) and indirect costs (such as work productivity) of HE, PROSPER should help confirm whether rifaximin-α 550 mg treatment represents a good use of economic resources.

**Trial registration:**

ClinicalTrials.gov identifier NCT02488993.

## Introduction

Hepatic encephalopathy (HE) is a brain dysfunction caused by liver insufficiency and/or portosystemic shunting, which manifests as a wide spectrum of neurological or psychiatric abnormalities [1]. It is now widely recognised as one of the most important severe complications of liver cirrhosis, along with conditions such as ascites and variceal bleeding [1]. HE severity is categorised using West Haven criteria from ‘minimal’ through grade 1 to 4 [1]. ‘Covert HE’ is defined as West Haven minimal and grade 1, and ‘overt HE’ is defined as West Haven grades 2 to 4 [1]. Overt HE occurs in 30–40% of patients with cirrhosis at some time during their clinical course [1]. HE increases the risk of mortality [2] and is one of the most debilitating complications of liver disease [1], negatively affecting the lives of both patients and caregivers [1, 3].

HE is also associated with a substantial economic burden, in terms of direct healthcare costs resulting from, for example, emergency room visits and hospitalisation [1, 4–11]. Although difficult to accurately determine, the indirect costs of HE (e.g., loss of work productivity, car accidents) also represent a substantial economic burden, which is likely to be further increased when the impact on caregivers is taken into account [3, 7, 12].

The neuropsychiatric symptoms of HE are thought to result from elevated blood levels of gut-derived neurotoxins (particularly ammonia), which enter the brain due to the inability of the cirrhotic liver to remove them from the blood [13]. Rifaximin-α 550 mg is a locally acting oral antibiotic that is minimally absorbed in the gut to reduce the effects of intestinal flora, including ammonia-producing species [14]. Its clinical activity may be due to its effects on the metabolic function of gut microbiota, rather than a change in relative bacterial abundance [15]. Rifaximin-α 550 mg is indicated in Europe for the reduction in recurrence of episodes of overt HE in patients aged ≥18 years [16]. In Australia, it has the same indication, but is restricted to where other treatments have failed or are contraindicated [17]. The efficacy of rifaximin-α 550 mg has been confirmed in a randomised controlled trial (RCT), in which rifaximin-α 550 mg twice daily (administered concomitantly with lactulose therapy in approximately 91% of patients) reduced the relative risks of recurrence of overt HE and HE-related hospitalisation by 58% and 50%, respectively, compared with placebo (absolute risk reductions: 24% and 9%, respectively) [18]. In a subsequent 2-year extension study, long-term treatment with rifaximin-α 550 mg (with concomitant lactulose in approximately 90% of patients) provided a continued reduction in the rate of HE-related and all-cause hospitalisation, without an increased rate of adverse events [19].

Although RCTs are essential in the clinical development of new treatments, they do not necessarily reflect clinical practice conditions. Typically, they are conducted in carefully defined patient populations using specific inclusion and exclusion criteria. By contrast, in clinical practice, patients are more diverse in terms of clinical characteristics than those recruited for RCTs. In addition, whereas RCTs employ rigid treatment protocols, in clinical practice treatment is tailored on a patient-by-patient basis. Consequently, ‘real-world’ studies are required to complement evidence from RCTs by determining how the efficacy of an agent translates into effectiveness in clinical practice. Since real-world studies have a higher likelihood of inherent bias than RCTs, it is important when designing a real-world study to eliminate as much bias as possible and minimise the impact of any remaining bias in the statistical analysis methodology employed. Real-world data are particularly useful for hard-to-reach patient populations (such as HE patients), for which clinical trial recruitment is challenging. Moreover, national reimbursement authorities are increasingly requesting real-world data to support evidence from clinical trials when examining the economic rationale for supporting new treatments [20, 21]. Real-world studies are therefore important both clinically and from a patient access perspective.

An important aspect of real-world studies is to assess patient- and caregiver-reported outcomes, since the effectiveness of a treatment is not only dependent on its efficacy and safety/tolerability, but also on its effects on the patient’s quality of life (QoL) and ability to function, and the associated impact on those who care for them. This is particularly true for chronic conditions, such as HE, where an agent’s effectiveness relies primarily on the patient being willing and able to be compliant with the treatment over the long term.

We here describe the design of the Prospective Real-world Outcomes Study of HE Patients’ Experience on Rifaximin-α 550 mg (PROSPER), which will evaluate the clinical effectiveness of rifaximin-α 550 mg and its impact on healthcare resources when used for the management of HE in routine clinical practice.

## Methods/design

PROSPER is a multinational, multicentre, observational study that will be conducted in secondary/tertiary care centres across Europe and Australia (ClinicalTrials.gov identifier: NCT02488993). Study site selection will be based on an initial feasibility assessment to evaluate the volume of patients seen and local expertise in HE at potential centres. The principal country for patient recruitment will be Denmark, where no ethical approval is required for observational studies. Ethical approval in other individual countries will be sought, where appropriate.

### Study design

PROSPER will be conducted under ‘real-world’ clinical practice conditions. Consequently, no changes to the management of HE patients will be made for the purposes of the study.

**Fig. 1:**
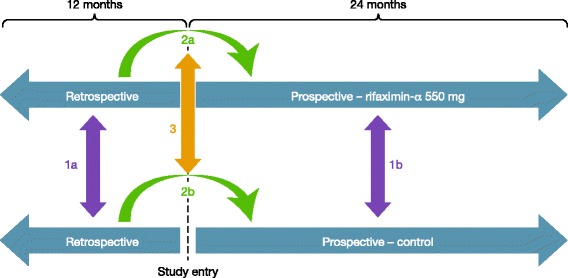
Study design

Once eligible patients have been recruited and consented to participate in the study, two data collection phases will be undertaken (Fig. 1). The first will be a retrospective phase, involving the review of the patients’ medical records and electronic hospital admissions data. Up to 12 months of data prior to study entry will be reviewed and collected. The level and nature of missing data will be assessed and accounted for in the statistical analysis plan, if necessary. The retrospective phase will be followed by a prospective phase, during which data will be collected from all patients either receiving rifaximin-α 550 mg (rifaximin-α 550 mg cohort) or not receiving rifaximin-α 550 mg (control cohort) from the point of study entry. Up to 24 months of data following study entry will be collected and analysed.

In order to minimise bias and the influence of confounding factors, the study outcomes will be compared between the treatment cohorts in three ways (Fig. 1). The first approach will initially involve the clinical characterisation of the study population, based on retrospective chart review (Fig. 1, comparison 1a), following which outcomes between the cohorts will be compared after adjustment of statistical methods on the basis of the findings from comparison 1a (Fig. 1, comparison 1b). Secondly, for each cohort individually, outcomes pre and post baseline will be compared (Fig. 1, comparisons 2a and 2b). Thirdly, the difference between the two cohorts in pre/post changes will be compared (Fig. 1, comparison 3).

**Fig. 2:**
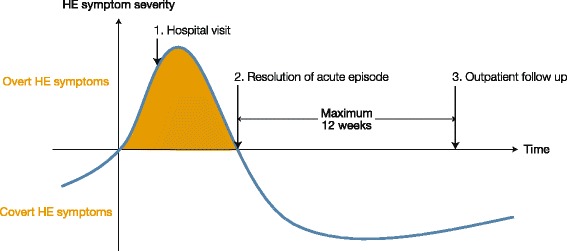
Timing of entry into study, relative to overt HE episode. HE, hepatic encephalopathy

Patients will be eligible to enrol during or following hospitalisation for the qualifying episode of overt HE (Fig. 2; timepoint 1). Patients will also be eligible to enter the study at the time of resolution of the qualifying episode of overt HE (Fig. 2; timepoint 2) or at a follow-up appointment, providing this is within 12 weeks of resolution of the qualifying episode of overt HE (Fig. 2; timepoint 3).

### Study population

Patients with cirrhosis and HE will be enrolled into PROSPER. The study will employ a limited number of inclusion and exclusion criteria in order to reflect the diversity of patients encountered in clinical practice (Table 1).

**Table 1:** Inclusion and exclusion criteria

Inclusion criteria	Exclusion criteria
• Diagnosis of cirrhosis	• West Haven score of ≥2 at study entry
• Age ≥ 18 years	• A mental health disorder that makes HE diagnosis questionable (e.g., dementia, psychosis)
• Enrolment within 12 weeks of resolution of an episode of overt HE associated with a hospital visit	• Prior treatment with rifaximin^a^ within 12 months prior to the qualifying overt HE episode
• Ability to provide informed consent	• Contraindications to the use of rifaximin-α 550 mg, as per the local Summary of Product Characteristics [16, 17]
• Clinical eligibility to receive rifaximin-α 550 mg, in the opinion of the participating physician, regardless of HE treatment actually received	

*HE* hepatic encephalopathy

^a^All types and dose strengths of rifaximin

### Study assessments

The study’s primary endpoint will be the HE- and liver-related hospitalisation rate and the resulting duration of hospitalisation (number of bed-days). Secondary endpoints will include the rate of all-cause hospitalisation and the resulting duration of hospitalisation (number of bed-days), mortality rate, and the number, duration and severity of HE episodes. Effects of treatment on the underlying liver disease will also be assessed as secondary endpoints using the Child-Turcotte-Pugh score, Model for End-stage Liver Disease (MELD) score or MELD-Na score (a modified MELD score including serum sodium [22]). In addition, the effectiveness of treatment will be assessed by the patient, caregiver and physician, using the Global Evaluation of Treatment Effectiveness (GETE) questionnaire. This consists of the single question ‘*How effective has [your treatment/the treatment] been in controlling [your/the patient’s] HE?*’, which is answered by choosing one of five responses: ‘*Complete control of HE*’, ‘*Marked improvement in HE*’, ‘*Limited improvement in HE*’, ‘*No appreciable change in HE*’ or ‘*Worsening of HE*’.

The safety of treatment with rifaximin-α 550 mg will be evaluated by assessing the frequency and nature of adverse events (AEs) and serious AEs. This will include classification of the severity of AEs and their relationship/causality in relation to medical treatment of HE.

The effects of treatment on health-related QoL will be assessed using the patient-reported Chronic Liver Disease Questionnaire (CLDQ) [23], the patient-reported Euroqol-5 Dimension-5 level (EQ-5D-5L) measure and the caregiver-reported proxy EQ-5D-5L:1 measure [24]. The CLDQ is a 29-item questionnaire assessing a wide range of physical, emotional and psychosocial QoL-related factors during the previous 2 weeks [23]. The EQ-5D-5L and proxy EQ-5D-5L are six-item questionnaires assessing the patient’s mobility, ability to self-care, ability to undertake usual activities, pain/discomfort, anxiety/depression and overall health status on the particular day in question [24].

Workplace productivity impairment will be assessed in patients using the Work Productivity and Activity Impairment Questionnaire: General Health V2.0 (WPAI:GH) and in caregivers using the Work Productivity and Activity Impairment Questionnaire: General Health Care Giving V2.0 (WPAI:GH-CG) [25]. These are six-item questionnaires assessing the effects of the patient’s health problems (WPAI:GH), or the effects of caregiving for the patient’s health problems (WPAI:GH-CG), on the individual’s ability to work and perform daily activities. A schedule for these assessments is shown in Table 2.

**Table 2:** Schedule of assessments

	Enrolment	1 month^a^	3 months^a^	6 months^a^	12 months^a^	18 months^a^	24 months^a^
Informed consent provided by patient and caregiver (note that caregiver consent is optional for patient study inclusion)	X						
Retrospective chart review (collection of demographic and medical history data)	X						
Ongoing clinical data, including clinical outcomes, laboratory measures, medication dose/schedule, resource utilisation, medication adherence^b^		X	X	X	X	X	X
GETE^c^		X		X			
CLDQ^b^	X				X		X
EQ-5D-5L (patient)^d^	X		X	X	X	X	X
EQ-5D-5L Proxy (caregiver)^d,e^	X		X	X	X	X	X
WPAI (patient)^c^	X		X	X	X	X	X
WPAI (caregiver)^c^	X		X	X	X	X	X

*CLDQ* Chronic Liver Disease Questionnaire, *EQ-5D-5 L* Euroqol-5 Dimension-5 level measure, *GETE* Global Evaluation of Treatment Effectiveness questionnaire, *WPAI* Work Productivity and Activity Impairment questionnaire

^a^Target dates only; flexible depending on patient response

^b^To be collected at all clinic visits but not at fixed time intervals; clinic visits will be scheduled by investigator and patient according to normal management and not defined by study protocol

^c^To be collected at the clinic visit closest to the indicated study date; no additional clinic visits to be scheduled for study participation

^d^To be requested for patient completion via paper submission or online portal or at clinic visit closest to the indicated date; clinic visit may not be required

^e^Australia, UK, Ireland, France and Germany, where the proxy response has been validated

### Sample size calculation

In observational studies where multivariable modelling is expected to be performed, the study should have at least 10 events for every variable included in the model. Therefore, in order to consider approximately 10 variables, 100 events would need to be observed in each of the study cohorts (patients receiving and not receiving rifaximin-α 550 mg). On the basis of an open-label maintenance study of rifaximin-α 550 mg in patients with HE [19], approximately 45% of patients would be expected to have experienced a hospitalisation within the first 12 months of follow-up. Assuming a dropout rate of 10%, approximately 247 patients would be required for each of the two cohorts, or 494 patients overall. Thus, a total study population of 550 would be sufficient to assess the primary endpoint of the study at an interim follow-up of 12 months, with additional precision following the full 24-month follow-up period.

### Statistical analysis

A primary aim of PROSPER will be to minimise any bias typically associated with observational studies. This will be achieved by the three-step comparative structure incorporated into the study’s design (as described above) and the associated statistical methodology. An overview of the statistical considerations is presented here.

The primary outcome of HE- and liver-associated hospitalisation rate will be analysed as a count, using either Poisson or negative binomial regression, depending on the level of observed dispersion. An offset term will be included to account for length of time eligible to experience a hospitalisation, accounting for mortality or other competing risk events. The primary outcome of duration of HE- and liver-associated hospitalisation (number of bed-days) will be analysed either as a continuous variable or a count, depending on the observed distribution. Methodologies such as median regression may be employed to account for the likelihood of right skew resulting from a small proportion of extended hospital stays. Comparison of the primary outcomes between treatment cohorts will adjust for key covariates using either multivariable regression analysis or a propensity score analysis (to be confirmed based on an exploratory review of the data). Due to the observational nature of the study, it is expected that some patients could change, discontinue or be non-compliant with therapy during the course of follow-up. These scenarios will be considered during statistical analysis and techniques that account for exposure to therapy will be applied, if applicable.

The rate of all-cause hospitalisation and the resulting duration of hospitalisation will be analysed in the same way as the primary endpoints. Numeric measures (e.g., CLDQ, EQ-5D-5L and GETE scores) will be analysed using a linear regression model, with longitudinal methodology to account for repeated measures, if applicable. Event-based variables and count data (e.g., AEs, HE episodes, mortality) will be analysed using Poisson or negative binomial regression, with offset terms to account for exposure time, length of time eligible to receive hospitalisation and competing risks (e.g., death); analyses will also adjust for underlying differences between cohorts (i.e., confounding factors). Time-to-event methods (e.g., Kaplan Meier analysis) will be used for variables such as time until death. Categorical demographic and clinical characteristics will be presented descriptively as frequency/percentage distribution and compared using a chi-squared test. Continuous demographic and clinical characteristics will be presented descriptively as means with 95% confidence intervals and compared using a Student’s *t*-test.

It is anticipated that additional analyses will include treatment adherence and switching, and the integration of survival data with QoL data, to assess quality-adjusted life-years (QALYs). Other exploratory analyses may also be conducted.

### Current status

PROSPER aims to enrol approximately 550 patients. Patient recruitment commenced in June 2015. An interim analysis will be performed after 12 months of data collection. Additional *ad hoc* and exploratory analyses may also occur during the course of data collection. The full dataset should be available for analysis by January 2020.

## Discussion

Data on the real-world effectiveness of secondary prophylaxis with rifaximin-α 550 mg and its impact on healthcare resource utilisation are currently limited. Although several studies have been conducted in the USA [7, 26–29], studies in Europe are scarce and none have yet been published in Australia. In the UK retrospective observational IMPRESS study, details of inpatient hospitalisations and hospital visits in the 12 months prior to and following initiation of rifaximin-α 550 mg treatment for HE were extracted from 11 NHS Trust electronic databases [4]. A total of 145 patients were evaluated (mean age 61 years; 61% male), 82% of whom were being treated with lactulose. A comparison of resource use in the 12 months pre- and post-initiation of rifaximin-α 550 mg revealed that there were significant reductions in the number of hospitalisations with overnight stay per patient (mean 2.7 vs. 1.7; *p* = 0.002), total number of bed days per inpatient (mean 31.7 vs. 16.4; *p* < 0.001) and number of critical care bed days per inpatient (mean 11.3 vs. 2.4; *p* = 0.017). The number of emergency room visits per patient also decreased but the difference was not statistically significant (mean 2.4 vs. 1.8; *p* = 0.099). Treatment with rifaximin-α 550 mg was generally well tolerated: three patients (2%) had adverse drug reactions and four (3%) developed *C. difficile* infection, but none of these patients discontinued treatment [4].

Another UK study specifically assessed the impact of rifaximin-α 550 mg treatment on healthcare resource utilisation using data from seven liver treatment centres [30]. Clinical, demographic and hospital admissions data from 326 patients were collected retrospectively for the time-periods 3, 6 and 12 months before and following initiation of rifaximin-α 550 mg treatment, and admission rates and hospital length of stay before and during therapy were compared. Rifaximin-α 550 mg treatment reduced the total length of stay in hospital by an estimated 31–53%, decreasing inpatient costs by £4858–6607 per patient per year. When the cost of treatment was taken into account (£3379 per patient per year), the estimated annual mean saving was £1480–3228 per patient [30].

Other data from Europe on the impact on healthcare resource utilisation are the results of cost-effectiveness modelling analyses conducted for the UK, Belgium, Sweden and the Netherlands [8–11], which applied country-specific costings to data derived from the original rifaximin-α 550 mg RCT [18] and open-label extension study [19]. The UK analysis found that the 5-year average cost of care for HE with rifaximin-α 550 mg plus lactulose was £22,971, a saving of £573 compared with the 5-year average cost of standard care (placebo plus lactulose) [8]. The corresponding values for benefit were 2.4 and 1.8 QALYs, respectively, representing a dominant base-case incremental cost-effectiveness ratio (ICER) over a 5-year horizon for rifaximin-α 550 mg. The positive impact of rifaximin-α 550 mg on healthcare costs was due to its reducing the rate of overt HE episodes, the likelihood of hospitalisation and hospital length of stay [8]. Similarly, beneficial cost-effectiveness results for rifaximin-α 550 mg treatment were demonstrated in modelling analyses conducted for Belgium and Sweden [9, 10]. The analysis conducted for the Netherlands incorporated indirect costs into the model, in terms of loss of work productivity and the costs of travelling to attend outpatient visits [11]. The time horizon was again 5 years, and costs and benefits were discounted at 4.0% and 1.5%, respectively. The total discounted and undiscounted 5-year costs for a patient treated with rifaximin-α 550 mg plus lactulose were €67,018 and €87,154, respectively, compared with €37,365 and €45,755, respectively, for a patient treated with placebo plus lactulose. Although the cost of lost productivity was higher for placebo plus lactulose than for rifaximin-α 550 mg plus lactulose (because of more frequent overt HE events), travel and informal costs were higher for rifaximin-α 550 mg plus lactulose than for placebo plus lactulose due to increased survival. The incremental health benefits for rifaximin-α 550 mg over a lifetime scenario were 0.93 QALYs (discounted) and 1.09 (undiscounted). Although the ICER amounted to €31,897 per QALY (discounted) and €38,027 per QALY (undiscounted), these values were well below the threshold recommended by the country’s Scientific Council for Government Policy (€80,000 per QALY) [11].

Although encouraging, these cost-effectiveness analyses were modelled using RCT data and their results therefore need to be confirmed with real-world evidence. PROSPER will address this need by providing valuable real-world information on the impact of rifaximin-α 550 mg treatment in Europe and Australia on both the direct costs (e.g., hospitalisation rate, duration of hospitalisation) and indirect costs (e.g., work productivity) of HE. PROSPER could also afford a better understanding of the burden and natural history of HE, and of variability in disease management between different countries and regions. Additionally, PROSPER is likely to provide the type of evidence required by payors and decision bodies to confirm whether rifaximin-α 550 mg treatment represents a good use of economic resources (as indicated by cost-effectiveness models), thereby ultimately allowing access to the right treatment for the right patients.

Since the findings and implications of real-world studies are often limited by bias, PROSPER has been specifically developed to minimise bias, in terms of both its design and the robust statistical methodology it employs. As such, it is broadly in line with the International Society for Pharmacoeconomics and Outcomes Research (ISPOR) Good Research Practices Taskforce guidelines, which state that the choice of study design may strengthen the ability to address potential biases and confounding in prospective observational studies, separately from the analytic and statistical approaches employed [20]. The details provided in the present article also address the guidelines’ recommendation that the reasoning behind all study design and analytic choices should be transparent and explained in the study protocol [20].

The ISPOR guidelines acknowledge the increasing importance of real-world studies in informing health policy decisions and highlight the consequent necessity for rigour and transparency when conducting such studies [20]. In addressing these concerns, it is anticipated that the findings of PROSPER will be widely applicable to clinical practice and may help inform future approaches to HE management.

## Conclusions

PROSPER will provide valuable real-world information on the effectiveness of rifaximin-α 550 mg in reducing the recurrence of HE, and its impact on the QoL and work productivity of patients and their caregivers. PROSPER will also help to confirm whether rifaximin-α 550 mg treatment is cost effective, by providing real-world information on its impact on both the direct and indirect costs of HE.

## Abbreviations

AE: Adverse event
CLDQ: Chronic Liver Disease Questionnaire
EQ-5D-5L: Euroqol-5 Dimension-5 level
GETE: Global Evaluation of Treatment Effectiveness
HE: Hepatic encephalopathy
ICER: Incremental cost-effectiveness ratio
ISPOR: International Society for Pharmacoeconomics and Outcomes Research
MELD: Model for End-stage Liver Disease
NHS: National Health Service
PROSPER: Prospective Real-world Outcomes Study of HE Patients’ Experience on Rifaximin-α 550 mg
QALY: Quality-adjusted life-year
QoL: Quality of life
RCT: Randomised controlled trial
WPAI:GH: Work Productivity and Activity Impairment Questionnaire: General Health V2.0
WPAI:GH-CG: Work Productivity and Activity Impairment Questionnaire: General Health Care Giving V2.0
